# Effects of ADC Nonlinearity on the Spurious Dynamic Range Performance of Compressed Sensing

**DOI:** 10.1155/2014/143693

**Published:** 2014-05-07

**Authors:** Rongzong Kang, Pengwu Tian, Hongyi Yu

**Affiliations:** Zhengzhou Information Science and Technology Institute, Zhengzhou 450002, China

## Abstract

Analog-to-information converter (AIC) plays an important role in the compressed sensing system; it has the potential to significantly extend the capabilities of conventional analog-to-digital converter. This paper evaluates the impact of AIC nonlinearity on the dynamic performance in practical compressed sensing system, which included the nonlinearity introduced by quantization as well as the circuit non-ideality. It presents intuitive yet quantitative insights into the harmonics of quantization output of AIC, and the effect of other AIC nonlinearity on the spurious dynamic range (SFDR) performance is also analyzed. The analysis and simulation results demonstrated that, compared with conventional ADC-based system, the measurement process decorrelates the input signal and the quantization error and alleviate the effect of other decorrelates of AIC, which results in a dramatic increase in spurious free dynamic range (SFDR).

## 1. Introduction 

Traditional approaches to acquiring and sampling signal are based on Nyquist sampling theory, which states that the sampling rate must be at least twice the maximum frequency of the input signal. The increasing demand for ADC with both wider bandwidth and higher quantization bits seems to contradict with each other. The new theory of compressed sensing (CS) [1, 2] introduced an alternative data acquisition framework, which states that CS enables the acquisition and recover of sparse signals in some transform domains at a rate proportional to their information content that is much below the Nyquist rate.

Analog-to-information converter (AIC) is designed to acquire samples at a lower rate for compressed sensing system, and various architectures have been proposed of the recent work in this area, such as the random demodulator sampling architecture [3], the modulated-wideband converter [4], and others [5–8]. However, in the view of practical hardware implementation, the basic components constitute an AIC consists of mixer, integrator/low passed filter and ADC, and so forth. Among these components, the ADC commonly has the lowest dynamic range; an A/D converter's deviation from its ideal “linear” performance is commonly characterized by the spurious-free dynamic range (SFDR) [9], which is defined as the difference in decibel, between the full-scale fundamental tone and the largest spurious harmonic component in the output spectrum. In order to make this notion precise, we will ignore the effects of any noise or nonlinearities from the other components except of ADC, since the SFDR of an AIC is typically dominated by the nonlinear process of ideal quantization and circuit-based (e.g., buffer, sample-and-hold) nonlinearities of ADC.

However there have been little literatures for characterizing and calculating the dynamic range performance of compressed sensing. In [10] a deterministic approach to dynamic range of a CS-based acquisition system is proposed, and the parameter of signal-to-quantization noise ratio is presented, whereas the dynamic parameter, that is, SFDR, is not considered. In [11] the quantization noise and dynamic range are considered for compressive imaging (CI) systems design and evaluate the quantization depth requirements for CI, while the quantization error and the SFDR performance of CS are still undiscussed. In [12] the impact of ADC nonlinearity in a mixed-signal CS system is studied, without considering the effect of ADC quantization error.

In this paper, we use an analytical approach couple with simulation results to formulate the SFDR performance of a compressed sensing system when considered with the quantization and nonlinearity of ADC. The background of compressed sensing is introduced firstly; then the power spectrum of quantization noise of AIC is analyzed numerically and the SFDR of ideal AIC-based system is derived. Furthermore, a detailed analysis of the other ADC nonlinear effects in SFDR performance of AIC-based system is presented. Finally, the behavioral simulations results are presented that clearly verify the accuracy of the analysis.

## 2. Background

### 2.1. Quantization-Limited SFDR of ADC-Based System

Quantization changes a sine wave from a smooth function to a staircase signal; due to this nonlinear effect, the output signal is composed of a large number of nonlinear distortion products. The most important contribution to the output distortion comes from the quantization process, because this is an inherently nonlinear process. In an ideal quantizer, suppose that there is no nonlinearity and noise exists except for the nonlinearity due to quantization. In this case, the spurious signal of ADC output is only produced by the quantization. When a sine wave is passed through the ideal quantizer, the Fourier series of the output signal leads to the closed-form expression for the magnitudes of the harmonic as [13]:

(1) $$\begin{array}{c} A_{p}=\delta_{p,1}A+\overset{\infty }{\underset{m=1}{\sum }}\frac{2}{m\pi }J_{p}\left(2m\pi A\right), \end{array}$$

where *A*_*p*_ is the output amplitude of the *p*th harmonic, *δ*_*p*,1_ is the Kronecker delta function, *A* is the input amplitude, and *J*_*p*_ is the *p*th-order Bessel function of the first kind. Although the largest harmonic is always located roughly at 2*πA* when the quantization levels are larger than 20, we consider the third harmonic as the largest and the power of the largest harmonic as a function of the number of bits. As a result, the quantization-limited SFDR performance of an ideal ADC-based system is approximated by [13]:

(2) $$\begin{array}{c} \text{SFDR}=8.07b+3.29\;\text{dB}. \end{array}$$

### 2.2. Nonlinear-Limited SFDR of ADC-Based System

Besides the nonlinearity produced by quantization, the circuit imperfections such as capacitor mismatches and finite opamp DC gains are considered. These nonlinearities of ADC would also influence the SFDR performance of the system. The simplest form of a nonlinear system is the memoryless power series, which is based on normal polynomials:

(3) $$\begin{array}{c} z=\overset{L}{\underset{i=0}{\sum }}a_{i}y^{i}, \end{array}$$

where *z* is the output signal, *y* is the input signal, and *L* is the order of the circuit nonlinearity. If the input signal is a single tone signal given by

(4) $$\begin{array}{c} y=A\cos\left(\omega t+\varphi_{0}\right), \end{array}$$

then the amplitudes of the harmonic terms can be computed from (3). However in the case of analog circuits, the order of a polynomial expression is mostly limited to third order, polynomial coefficients of the 4th order and higher, and the nonlinearity caused by saturation at full scale are neglected.

When substituting (4) into (3), we get

(5) z=∑i=0Laiyi=a1[Acos⁡(ωt+φ0)]+a2[Acos⁡(ωt+φ0)]2 +a3[Acos⁡(ωt+φ0)]3,z≅a1cos⁡(ωt+φ0)+12a2A2[cos⁡(2ωt+2φ0)] +14a3A2[cos⁡(3ωt+3φ0)]2.

The specific relation between polynomial coefficients and harmonic power can be expressed in general [14]. The same analysis can be done when the input signal is supposed to be two tone signals; it will cause the production of more terms, the specific terms harmonics, and intermodulation. Furthermore, the dynamic range performance of ADCs is specified in terms of one-tone and two-tone SFDR [15].

While in practice, tests which have been developed to measure the performance mostly rely on Fourier analysis using discrete Fourier transform (DFT) and the fast Fourier transform (FFT). The input analog signal is first sampled at Nyquist rate; the harmonics and intermodulation distortion are calculated through the input signal spectrum, which is estimated from the time-domain samples with nonlinear distortion via DFT. However the DFT-based method needs to avoid the leakage of the input frequency and the number of periods of the input waveform in the sample record should not be a nonprime integer submultiple of the record length, further the ADC needs to have a high resolution, which limits the maximum achievable sampling rate.

As an alternative solution to high-speed ADCs, AIC-based system enables high resolution at high frequencies while only using low frequency, sub-Nyquist ADCs [3–8]. In this work, we investigate the effect of nonlinearity induced by quantization and other circuit's nonidealities of ADC on the AIC-based system and examine the SFDR performance in the presence of these nonlinearities.

### 2.3. Analog-to-Information Converter (AIC)

**Figure 1:**
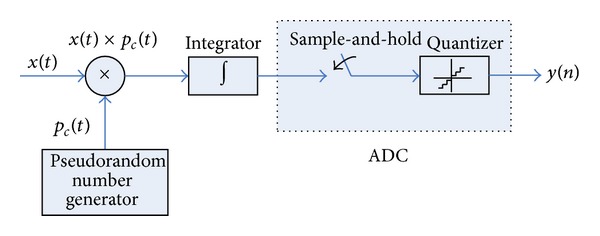
Block diagram of the random demodulator.

There have been many theoretical discussions on AIC system in the literature [3–8], in this work, the block diagram of a typical AIC implementation [3] called the random demodulator shown in Figure 1 is considered to compare with the conventional ADCs. In this architecture, the input signal *x*(*t*) is mixed by a different pseudorandom number *p*_*c*_(*t*) waveform; then the mixer output is integrated over a time period of 1/*M*. Finally, the integrator outputs are sampled and quantized, by a traditional integrate-and-dump ADC at *M* Hz.

Note that this AIC architecture employs sub-Nyquist rate ADCs, and the input signal is mixed with the PN sequence and sent to integrator before sampling. As a result, the spectrum of the signal sent to the ADC is relatively flat within the filter pass band, and then the harmonic and intermodulation energy due to the nonlinearity of ADC is spread along the signal bandwidth rather than concentrate on a few tones, which can lead to a better SFDR performance after reconstruction. In the following section we present our framework for investigating the impacts of nonlinearity caused by quantization and other circuits induced on the SFDR performance of AIC-based system.

## 3. SFDR Performance of AIC-Based System

### 3.1. Quantization-Limited SFDR of AIC-Based System

In an ideal case the dynamic range performance is mainly limited by quantization error; the spectra of the AIC quantization output is analyzed in this section.

As we know, the time-domain expression of the measuring process of AIC is given by

(6) $$\begin{array}{c} y_{i}=\langle x,\phi_{i}\rangle =\overset{N}{\underset{j=1}{\sum }}\phi_{ij}x_{j}, \end{array}$$

where *ϕ*_*ij*_ is the element of measurement matrix and *x*_*j*_ is the element of the input signal. Suppose that the measurement matrix is sub-Gaussian random matrix; then the element *ϕ*_*ij*_ is independent centered sub-Gaussian random variables with variance 1/*M*, given *S*_*i*,*j*_ = *ϕ*_*i*,*j*_ *x*_*j*_; then

(7) $$\begin{array}{c} y_{i}=\overset{N}{\underset{j=1}{\sum }}S_{ij}. \end{array}$$

Then we can get the mean and variance of *S*_*i*,*j*_:

(8) E[Si,j]=E[ϕi,jxj]=xjE[ϕi,j]=0.D[Si,j]=E[Si,j2]=xj2E[ϕi,j2]=xj2M.

According to the central limit theorem, when *N* → *∞*, the *y*_*i*_ subject to Gaussian distribution with mean 0 and variance ∑_*j*=1_^*N*^(*x*_*j*_^2^/*M*) = ||X||_2_^2^/*M*.

As we know that when the input signal subjected to Gaussian distribution with mean 0, then the relation between autocorrelation function *R*_*e*_(*m*) of quantization error and input signal can be expressed as follows:

(9) $$\begin{array}{c} R_{e}\left(m\right)=\frac{\Delta^{2}}{2\pi^{2}}\overset{\infty }{\underset{k=1}{\sum }}\frac{1}{k^{2}}\exp\left[-4\pi^{2}\frac{\sigma^{2}}{\Delta^{2}}k^{2}\left(1-r_{y}\left(m\right)\right)\right], \end{array}$$

where Δ is the quantization step size and *σ* ^2^ is the variance of the input signal. *r*_*y*_(*m*) = *R*_*y*_(*m*)/*R*_*y*_(0) represents the normalized autocorrelation function. While the autocorrelation function of the measurement value can be expressed as

(10) Ry(m)=E[yiyi+m]=E[∑j=1Nϕi,jxj∑k=1Nϕi+m,kxk]=E[∑j=1N ∑k=1Nϕi,jϕi+m,kxjxk]=∑j=1N ∑k=1NE[ϕi,jϕi+m,k]xjxk.

Because the element of the measurement matrix is independent, then *R*_*y*_(0) = ||X||_2_^2^/*M*, when *j* = *k* and *m* = 0, for others  *R*_*y*_(*m*)  equal to 0, so normalized autocorrelation function is

(11) $$\begin{array}{c} r_{y}\left(m\right)=\{\begin{array}{cc} 1, & m=0,\\ 0, & \text{else}. \end{array} \end{array}$$

So, the autocorrelation function  *R*_*e*_(*m*) of quantization error is

(12) $$\begin{array}{c} R_{e}\left(m\right)=\{\begin{array}{cc} \frac{\Delta^{2}}{2\pi^{2}}\overset{\infty }{\underset{k=1}{\sum }}\frac{1}{k^{2}}, & m=0,\\ \frac{\Delta^{2}}{2\pi^{2}}\overset{\infty }{\underset{k=1}{\sum }}\frac{1}{k^{2}}\exp\left[-4\pi^{2}\frac{\sigma^{2}}{\Delta^{2}}k^{2}\right], & \text{else}, \end{array} \end{array}$$

where  *σ*/Δ ≥ 1, and when  *σ*/Δ = 1 and  *m* ≠ 0,

(13) $$\begin{array}{c} R_{e}\left(m\right)=\frac{\Delta^{2}}{2\pi^{2}}\left[\frac{e^{-4\pi^{2}}}{1}+\frac{e^{-16\pi^{2}}}{4}+\frac{e^{-36\pi^{2}}}{9}+\cdots \right]. \end{array}$$

For *e* ^−4*π*^2^^ ≈ 7 × 10^−18^, *e* ^−16*π*^2^^ ≈ 2 × 10^−69^, we get *R*_*e*_(*m*) ≈ 0, when *m* ≠ 0, and *R*_*e*_(0) = (Δ^2^/2*π* ^2^)∑_*k*=1_^*∞*^(1/*k* ^2^) = (Δ^2^/12).

From the above analysis, we know that *R*_*e*_(*m*) is approximated to *δ* function, and according to the Fourier transform relationship between power spectrum and autocorrelation function, the power spectrum of quantization noise is white noise spectrum. As a result, the spurious energy due to the quantization effect of ADC is spread to the whole bandwidth, and we can get a better SFDR performance of AIC-based system compared with the conventional ADC-based system.

### 3.2. Nonlinear-Limited SFDR of AIC-Based System

Compared with the analysis of the conventional ADC-based system, in AIC-based system, the input signal goes through random projection, filtering, and sampling.

A signal *x* can be viewed as a  *N* × 1  column vector in  *R* ^*N*^  with elements *x*[*n*], *n* = 1,2,…, *N*. Let the matrix  Ψ = [*ψ*_1_, *ψ*_2_,…*ψ*_*N*_]  have columns which form a basis of vectors in *R* ^*N*^. And then, any signal *x* can be expressed as

(14) $$\begin{array}{c} x=\overset{N}{\underset{i=1}{\sum }}s_{i}\psi_{i}\;\text{or}\;x=\Psi s, \end{array}$$

where *s* is the  *N* × 1  column vector of weighting coefficients  *s*_*i*_ = 〈*x*, *ψ*_*i*_〉.

Consider a generalized linear measurement process of a signal  *x*  which is *K*-sparse. When we say that *x* is *K*-sparse, we mean that it is well reconstructed or approximated by a linear combination of just  *K*  basis vectors from Ψ, with *K* ≪ *N*. That is, there are only  *K*  of the  *s*_*i*_  in (1) that are nonzero and  (*N* − *K*)  are zero. Let  Φ  be an  *M* × *N*  measurement matrix,  *M* ≪ *N*  where the rows of  Φ  are incoherent with the columns of Ψ. The incoherent measurements can be obtained by computing  *M* inner products between  *x*  and the rows of  Φ  as in  *y*_*j*_ = 〈*x*, *ϕ*_*j*_〉. It can also be expressed as

(15) $$\begin{array}{c} y=\Phi x=\Phi \Psi s=\Theta s, \end{array}$$

where  Θ : = ΦΨ  is a  *M* × *N* matrix. It is proved that  Φ  does not depend on the signal *x* and it can be constructed as a random matrix such as Gaussian matrix.

Then according to the nonlinearity model of ADC, we substitute the transform-domain samples into (3), and then we can get the measurement output of the AIC with nonlinear effect as follows:

(16) $$\begin{array}{c} z=\overset{L}{\underset{i=0}{\sum }}a_{i}y^{i}=a_{1}\left[\Phi \Psi s\right]+a_{2}{\left[\Phi \Psi s\right]}^{2}+a_{3}{\left[\Phi \Psi s\right]}^{3}. \end{array}$$

### 3.3. Reconstruction of Frequency Sparse Signal

After quantization and sampling of ADC, we get the measurement in discrete values, in order to evaluate the SFDR performance of the AIC-based system, we need to compute the spectrum of the reconstruction signal. So, in this section, we frame the reconstruction problem for the AIC-based system with the nonlinearity effect.

Furthermore, the spectrum of the input signal is estimated from the measurement value  *z*  with nonlinear distortion via solving the following optimization problem:

(17) $$\begin{array}{c} \hat{s}=\arg\min{||s||}_{1}\;\;\text{s}.\text{t}.\;{||y-\Phi \Psi s||}_{2}\leq \varepsilon_{n}+\varepsilon_{d}, \end{array}$$

where  *ε*_*n*_ is the error due to the noise and  *ε*_*d*_  is the error due to the nonlinear distortion.

Up to now, there are many mature algorithms to resolve this convex optimization problem, including interior-point algorithms [16, 17], gradient projection [18], iterative thresholding [19, 20], and greedy approaches such as orthogonal matching pursuit (OMP) [21, 22]. Here we use the algorithm of basis pursuit with denoising [23] to resolve the reconstruction problem for evaluation of SFDR performance of AIC-based system.

## 4. Simulation Results

**Figure 2:**
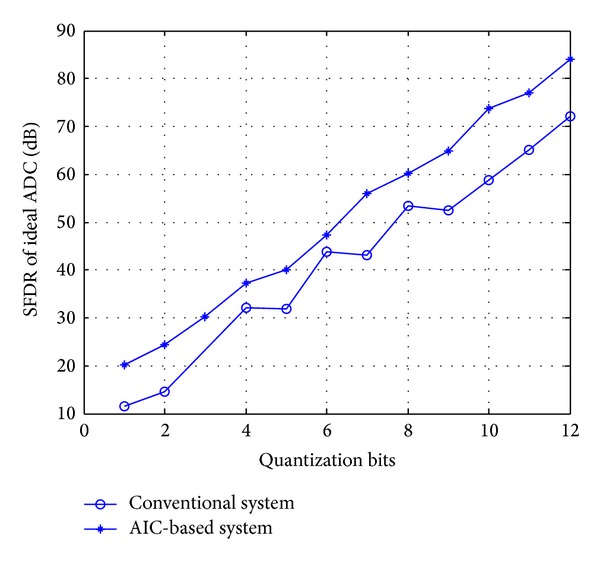
SFDR performance of AIC-based system and ADC-based with ideal ADC.

Figure 2 shows the SFDR performance of conventional ADC-based system and AIC-based system of ideal ADC with a single sinusoidal input for different quantization bits.  Φ is set to an  *M* × *N* Gaussian random measurement; *M* = 256, and *N* = 1024  . The input signal frequency is *f*_0_ = 64 Hz, *f*_*s*_ = (5∗64 − 1) Hz, and use BPDN [23] as the reconstruction algorithm. Every measurement was repeated 300 times to test the reproducibility.

As shown in Figure 2, the SFDR performance of AIC-based system outperforms that of conventional ADC-based system. That is because in the conventional ADC-based system, noise spectrum of sinusoid signals consists of discrete components, and the harmonic is concentrated in the odd multiple of its fundamental frequency, while in the AIC-based system the spectrum of quantization error is uniformly distributed. However the total quantization noise power represented by the area under the noise spectrum is approximately equal to Δ^2^/12, for AIC-based system the spurious energy is spread along the whole signal bandwidth; then each harmonic of the quantization error is thereby pulled downward into a more dense portion of the noise spectrum leading to increasing in SFDR performance. The observation from this simulation was intuitively illustrated in Figure 2.

**Figure 3:**
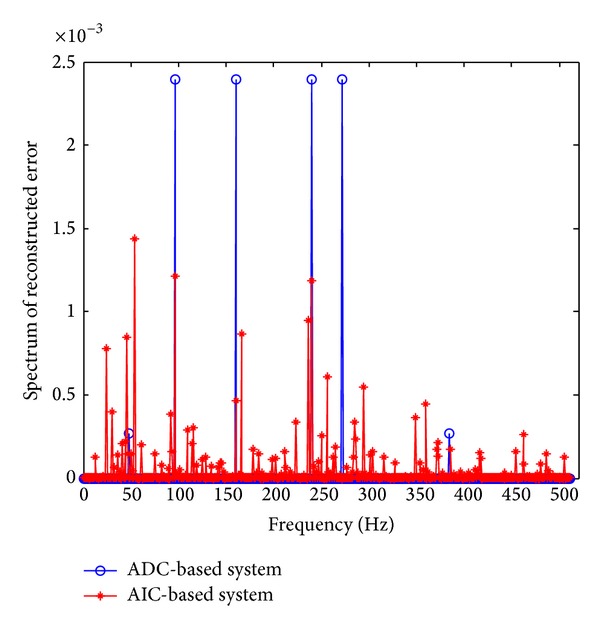
Spectrum of reconstruction error comparison between the conventional ADC-based system and the AIC-based system.

Figure 3 shows a snapshot of the single-tone reconstructed error spectrum for conventional system and CS-based system. The second-order *a*_2_ = 0.1  and third-order distortion coefficients *a*_3_ = 0.1. As we can see, in the conventional ADC-based system, the spurious harmonic due to the ADC nonlinearity concentrates on the multiple of fundamental frequency, whereas, in the CS-based system, the spurious energy is spread along the whole signal bandwidth. Meanwhile the amplitude of the spurious harmonic of AIC-based is lower than that of ADC-based system.

**Figure 4:**
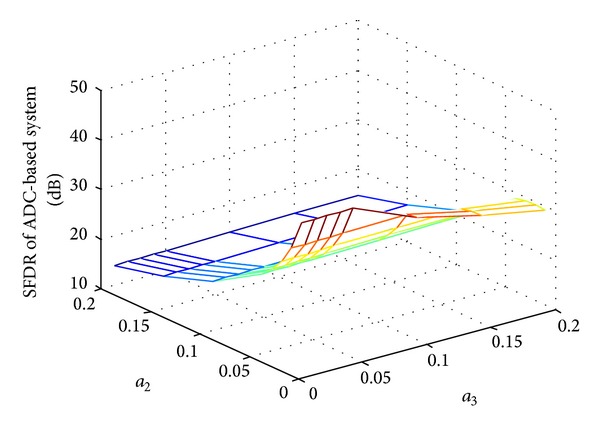
SFDR performance of conventional ADC-based system with other nonlinear effects.

**Figure 5:**
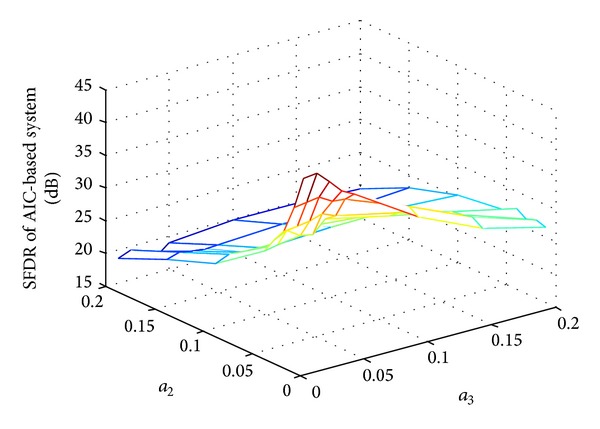
SFDR performance of for AIC-based system with other nonlinear effects.

Figures 4 and 5 show the SFDR performance of ADC-based system and AIC-based system for two-tone input with quantization and different nonlinear distortion coefficients.  *f*_1_ = 16 Hz, *f*_2_ = 256 Hz, *f*_*s*_ = 1024 Hz, and quantization bits *N* = 4. The reconstruction algorithm and other simulation conditions are set the same as in Figure 2.

As we can see, both of the SFDR performances decrease when nonlinear distortion becomes large with the second- and third-order distortion coefficients increase. The simulation results also indicate that the second-order distortion influences the SFDR performance more seriously than that of the third-order distortion.

Comparing the results of Figure 4 with Figure 5, we can see that the SFDR performance of AIC-based system outperforms that of the ADC-based system when introducing the nonlinearity with both the quantization and circuit nonideality. This is because the randomization in AIC-based system changes the distribution of the error power from ADC nonlinear distortion; the signals sent to ADCs in the conventional Nyquist sampling architecture are original sinusoid signals, whereas those in the AIC-based system have relatively flat spectrum. As a result, by spreading the spurious energy along the signal bandwidth, the CS randomization relaxes the requirement on the ADC SFDR specification.

## 5. Conclusions

In this paper, we compare the SFDR performance of AIC-based system and conventional ADC-based system when considering both nonlinearity due to quantization and other circuit nonideality of ADC. We demonstrate that the quantization noise of AIC is spectrally white and uniformly distributed, and the quantization harmonics of AIC-based system is spread to the whole bandwidth, which means an improvement of SFDR performance. We show that AIC-based systems are less sensitive to the nonlinearity of ADC because of the CS randomization, which provides improvement of SFDR performance compared with conventional ADC-based system. Our results suggest that the second- and third-order distortion coefficients and quantization bits are the main factors that affect the SFDR performance of compressed sensing. The results presented in this paper can also be easily extended to the case when the signals input to AIC are multisinusoids.
